# Excessive Media Consumption About COVID-19 is Associated With Increased State Anxiety: Outcomes of a Large Online Survey in Russia

**DOI:** 10.2196/20955

**Published:** 2020-09-11

**Authors:** Nikita A Nekliudov, Oleg Blyuss, Ka Yan Cheung, Loukia Petrou, Jon Genuneit, Nikita Sushentsev, Anna Levadnaya, Pasquale Comberiati, John O Warner, Gareth Tudor-Williams, Martin Teufel, Matthew Greenhawt, Audrey DunnGalvin, Daniel Munblit

**Affiliations:** 1 Department of Paediatrics and Paediatric Infectious Diseases Institute of Child’s Health Sechenov First Moscow State Medical University (Sechenov University) Moscow Russian Federation; 2 School of Physics, Astronomy and Mathematics University of Hertfordshire Hatfield United Kingdom; 3 Inflammation, Repair and Development Section, National Heart and Lung Institute Faculty of Medicine Imperial College London London United Kingdom; 4 Department of Bioengineering Imperial College London London United Kingdom; 5 Pediatric Epidemiology, Department of Pediatrics Medical Faculty Leipzig University Leipzig Germany; 6 Department of Radiology Addenbrooke’s Hospital and University of Cambridge Cambridge United Kingdom; 7 National Medical Research Center for Obstetrics, Gynecology and Perinatology Moscow Russian Federation; 8 Department of Clinical and Experimental Medicine Section of Pediatrics University of Pisa Pisa Italy; 9 Department of Infectious Disease Faculty of Medicine Imperial College London London United Kingdom; 10 Clinic for Psychosomatic Medicine and Psychotherapy LVR University Hospital University of Duisburg-Essen Essen Germany; 11 Department of Pediatrics, Section of Allergy/Immunology Children’s Hospital Colorado University of Colorado School of Medicine Aurora, CO United States; 12 Applied Psychology and Paediatrics and Child Health University College Cork Cork Ireland; 13 Solov’ev Research and Clinical Center for Neuropsychiatry Moscow Russian Federation

**Keywords:** anxiety, COVID-19, media consumption, SARS-CoV-2, STAI, state anxiety, trait anxiety, trust to government, trust, mental health, social media, survey

## Abstract

**Background:**

The COVID-19 pandemic has potentially had a negative impact on the mental health and well-being of individuals and families. Anxiety levels and risk factors within particular populations are poorly described.

**Objective:**

This study aims to evaluate confidence, understanding, trust, concerns, and levels of anxiety during the COVID-19 pandemic in the general population and assess risk factors for increased anxiety.

**Methods:**

We launched a cross-sectional online survey of a large Russian population between April 6 and 15, 2020, using multiple social media platforms. A set of questions targeted confidence, understanding, trust, and concerns in respondents. The State-Trait Anxiety Inventory was used to measure anxiety. Multiple linear regressions were used to model predictors of COVID-19–related anxiety.

**Results:**

The survey was completed by 23,756 out of 53,966 (44.0% response rate) unique visitors; of which, 21,364 were residing in 62 areas of Russia. State Anxiety Scale (S-Anxiety) scores were higher than Trait Anxiety Scale scores across all regions of Russia (median S-Anxiety score 52, IQR 44-60), exceeding published norms. Time spent following news on COVID-19 was strongly associated with an increased S-Anxiety adjusted for baseline anxiety level. One to two hours spent reading COVID-19 news was associated with a 5.46 (95% CI 5.03-5.90) point difference, 2-3 hours with a 7.06 (95% CI 6.37-7.74) point difference, and more than three hours with an 8.65 (95% CI 7.82-9.47) point difference, all compared to less than 30 minutes per day. Job loss during the pandemic was another important factor associated with higher S-Anxiety scores (3.95, 95% CI 3.31-4.58). Despite survey respondents reporting high confidence in information regarding COVID-19 as well as an understanding of health care guidance, they reported low overall trust in state and local authorities, and perception of country readiness.

**Conclusions:**

Among Russian respondents from multiple social media platforms, there was evidence of higher levels of state anxiety associated with recent job loss and increased news consumption, as well as lower than expected trust in government agencies. These findings can help inform the development of key public health messages to help reduce anxiety and raise perceived trust in governmental response to this current national emergency. Using a similar methodology, comparative surveys are ongoing in other national populations.

## Introduction

In December 2019, the first patients with pneumonia of unknown cause were linked to a seafood wholesale market in Wuhan, China [1]. This is generally recognized as the beginning of a previously unknown beta-coronavirus pandemic related to the SARS-CoV-2 virus, which has subsequently spread worldwide. This COVID-19 pandemic has resulted in dramatic changes to normal life in many countries, leading to disruptions of social and economic functioning comparable to the impact of the Spanish flu pandemic of 1918.

COVID-19 illness has rapidly spread, producing high numbers of fatalities in the absence of proven pharmacologic treatments and vaccines, [2] and lag time in application of testing, contact tracing, and mass quarantine measures. Concomitantly, there has been a rise in reported mental health problems such as fear, anxiety, depression, and sleep problems among different subgroups worldwide [3,4]. A recent UK survey and Ipsos MORI poll showed public concerns about the effect of social isolation or social distancing on well-being; increased anxiety, depression, stress, and other negative feelings; and concerns related to current and potential future financial difficulties [5].

A recently published Lancet Psychiatry position paper highlighted the need to collect high-quality data on the mental health effects of the COVID-19 pandemic across the whole population as a top priority [5] requiring global action. The World Health Organization (WHO) has outlined research priorities for containing COVID-19 and supporting those affected. A March 26, 2020, press briefing specifically addressed the pandemic’s effect on mental health: *“With the disruptive effects of COVID-19 – including social distancing – currently dominating our daily lives, it is important that we...are mindful of and sensitive to the unique mental health needs of those we care for. Our anxiety and fears should be...better understood and addressed”* [6]*.* Strategies advocating physical or social distancing have become central to pandemic control in many countries, but to be effective, these require universal adoption within society, which has been variable internationally. Quality of communication can be quite impactful on personal psychology and behaviors during health emergencies. The WHO Director-General has cautioned about an *infodemic* of misinformation in online platforms, which may negatively impact how society is perceiving and responding to the COVID-19 outbreak.

The aim of this study is to try to measure associations between COVID-19 perception and anxiety from an international perspective to address unmet needs in the field. We hypothesized that there would be increased COVID-19–related state anxiety (driven by pandemic-specific events affecting individuals) compared with pre-existing levels of trait anxiety, which likely varies depending on the country surveyed. Herein we present the initial findings on levels of state and trait anxiety (and their determinants) in a large sample of people residing in Russia during the COVID-19 pandemic, a previously poorly described region with respect to global mental health concerns.

## Methods

### Study Design and Population

A cross-sectional open 160-item online survey was conducted between April 6 and 15, 2020, timed to follow the Russian government’s announcement of a “stay-at-home” order through April 30 [7]. The survey was promoted on three social media platforms (VK, Facebook, and Instagram) via influencers, a popular Russian search engine (Yandex), and the Russian internet media portal (Meduza). We used nonprobability sampling [8] through social media to allow for rapid data collection, which was particularly important at the peak of the lockdown period. The survey was pretested with members of the public that had no role in the questionnaire design to ensure good understandability and identify discrepancies in wording as well as missing facets that may have been previously overlooked. No registration was required to access the survey. This paper is compliant with the CHERRIES (Checklist for Reporting Results of Internet E-Surveys) [9].

### Ethics Statement

The study was reviewed and approved by Sechenov University Ethics Committee on April 2, 2020. Participants were informed at the outset that by completing and submitting their responses to the online survey, they were consenting to voluntary participation in a research study of attitudes and behaviors surrounding COVID-19. The survey settings and web analytics were accessible only to authors DM and NAN. At the end of the survey, participants were asked to provide a limited amount of personal data (email) that participants agreed could be held on the research database. This request was optional and did not have any impact on survey completion.

### Survey Questionnaire

For the purpose of this survey, we modified and further adapted a questionnaire developed by author MT at the University of Duisburg-Essen, Clinic for Psychosomatic Medicine and Psychotherapy, LVR University Hospital Essen [10]. A single master survey was developed for adaptation, translation according to WHO protocol, and similar dissemination in other countries to provide a comparative analyses of COVID-19 risk factors for mental health across countries, with particular attention to the impact of information.

The survey consisted of several modules, assessing basic demographic information; socioeconomic status; employment; living conditions; health status; medications intake; time following news on COVID-19; confidence in and understanding of information; trust of state authorities; trust of local authorities; worry, concern, or adverse expectations; perception of risk; personal protection measures; and behavioral aspects.

The health status of each participant was categorized, taking into account previous data on chronic conditions’ impact on mental health and up-to-date evidence on the risk factors for mortality from COVID-19 infection. People with depression or cardiological and respiratory conditions were among the main potential determinants of anxiety; cardiological and respiratory conditions are known risk factors for mortality from COVID-19 [11], whereas depression is known to have a detrimental association with anxiety [12]. If more than one of these factors were present in an individual, we considered this a higher risk and subcategorized respondents into another subgroup. Participants reporting oncological conditions or HIV and diabetes, renal, or hepatic problems were combined into subcategories due to a limited number of people reporting these chronic diseases.

Four psychometric measures, previously validated in the Russian population, were included: the State-Trait Anxiety Inventory (STAI), Big Five Inventory-10, General Self-Efficacy Scale, and the Patient Health Questionnaire. We applied branching logic where it was justified to spare the users’ time while providing their responses. The survey was partitioned into 7 pages, including the introduction page containing general information about the survey. The estimated time to complete the survey did not exceed 20 minutes.

### State and Trait Anxiety Inventory

The STAI was used to measure self-reported presence and severity of current symptoms of anxiety and a generalized propensity to be anxious [13]. The STAI has been previously validated for use in the Russian population and is freely available in the Russian language [14]. The index consists of two subscales. The State Anxiety Scale (S-Anxiety) measures current anxiety in the moment, assessing subjective feelings of apprehension, tension, nervousness, worry, and activation or arousal of the autonomic nervous system. The Trait Anxiety Scale (T-Anxiety) evaluates relatively stable aspects of *anxiety proneness*, including general states of calmness, confidence, and security [13]. The range of scores for each subtest is 20-80, the higher score indicating greater anxiety.

### Confidence, Understanding, Trust, and Concerns

Fifteen ad-hoc designed questions assessed self-perceived confidence in information and understanding (feeling informed about COVID-19 and understanding the guidance from health care authorities); trust in state and local authorities, and country readiness for the pandemic; governmental measures (whether respondents consider measures excessive or not); worry, concern, or adverse outcome expectations, including potential consequences to the individual and the country. Respondents were provided with a 9-point Likert scale, where 1 represented *complete disagreement* and 9 *complete agreement* with a given statement.

### Statistical Analysis

Descriptive statistics were used for baseline characteristics of responders including sociodemographics and scores of psychometric tools. Multiple linear regressions were used to model the association of potential variables of interest with the results of psychometric tools. Additional multiple logistic regression was also performed with the dichotomized S-Anxiety score as the outcome using the cutoff of 45. Multiple analyses were adjusted for the available baseline characteristics. In line with suggested recommendations [15], the results were adjusted for the multiple comparisons using a Bonferroni correction, which resulted in an alpha value threshold of *P*=.001 being used for statistical significance.

Distributions of the tests’ results were assessed with density plots and box and whisker plots. All the analyses were performed using R version 3.6.3 (The R Foundation for Statistical Computing). Maps of state and trait anxiety across regions of Russia were plotted using the “ssplot” library. Two-sided *P* values were reported for all statistical tests.

## Results

### Participants

The survey link was accessed 57,877 times. The number of unique visitors was 53,966, of which 42,643 (79.0% participation rate) gave their consent to participate and accessed the first survey page; 23,756 out of 42,643 (55.7% completion rate) users completed the questionnaire. The response rate was 44.0% with 23,756 responses from 53,966 unique visitors. Unique users were identified using cookies set at the top-level domain, the expiration date was preset to 1 year. The view ratio could not be calculated due to the variety of traffic sources used for survey distribution. The presets did not allow users to send incomplete questionnaires, so those were not present in the exported tabulated data set. There was no specific cutoff that would exclude a questionnaire from the analysis.

The demographic characteristics of the study participants are summarized in Table 1. Out of 23,756 respondents, 21,364 were residing in Russia at the time of survey completion; data from all of the latter were included in the analyses (median age 32 years, IQR 28-36; range 18-82 years). Out of 21,364 respondents, 18,609 (87.1%) were female, 14,752 (68.2%) were married or in a relationship, 14,371 (67.3%) had children younger than 18 years, and 4.4% (n=933) were expecting a child at the time of survey completion. Of the 21,364 participants, 53% (n=11,450) reported various chronic conditions and 3789 (17.7%) were current smokers.

The 21,364 respondents were equally distributed between the capital (n=7468, 35.0%), large cities (n=6348, 29.7%), and smaller localities (n=7548, 35.2%). A total 62 areas of Russia were represented with at least 40 respondents from each location. The population was highly educated with 17,688 (82.8%) having a higher degree and a further 1635 (7.7%) studying at university. There were 1648 (7.7%) respondents that lost their jobs due to the COVID-19 pandemic. There were 19,589 (91.7%) respondents who were not related to the health care profession and did not study medicine.

**Table 1:** Sociodemographic characteristics of survey respondents residing in the Russian Federation at the time of the COVID-19 pandemic (N=21,364).

Characteristics		Participants
Sex (female), n (%)		18,609 (87.1)
Age (years), median (IQR)		32 (28-36)
**Age (years), range**		18-82
	18-25, n (%)	2991 (14)
	26-35, n (%)	12,893 (60.3)
	36-45, n (%)	4418 (20.7)
	≥46, n (%)	1062 (5)
Marital status, married or in relationship, n (%)		14,752 (68.2)
Have children younger than 18 years, n (%)		14,371 (67.3)
Expecting a child, n (%)		933 (4.4)
**City of residence, n (%)**		
	Capital	7468 (35.0)
	Large city (over 500,000 inhabitants)	6348 (29.7)
	Smaller cities/towns	7548 (35.2)
**Education status, n (%)**		
	PhD	547 (2.6)
	More than one degree	1458 (6.8)
	Master’s degree	4362 (20.4)
	Bachelor’s degree	11,321 (53.0)
	Higher education in progress	1635 (7.7)
	Vocational school	1507 (7.0)
	School	412 (1.9)
	Other	122 (0.6)
**Income (R), n (%)**		
	Decline to answer	928 (4.3)
	<20,000	4377 (20.5)
	20,000-34,999	5308 (24.8)
	35,000-69,999	6012 (28.1)
	70,000-99,999	2412 (11.3)
	100,000-149,999	1351 (6.3)
	≥150,000	976 (4.6)
**Chronic medical conditions, n (%)**		
	No	9603 (44.9)
	Decline to answer	311 (1.5)
	Depression and cardiological or respiratory	247 (1.2)
	Depression or neurological	523 (2.4)
	Allergies (food allergy/allergic rhinitis) or dermatological (eczema/psoriasis)	2283 (10.7)
	Cardiological	742 (3.5)
	Cardiological and respiratory	65 (0.3)
	Renal/hepatic/diabetes	705 (3.3)
	Oncology/HIV	269 (1.3)
	Other	6438 (30.1)
	Respiratory	178 (0.8)
Neuroleptics/antidepressant use, n (%)		832 (3.9)
**Time spent on reading COVID-19 news, n (%)**		
	Decline to answer	39 (0.2)
	Do not follow	335 (1.6)
	Do not follow but they find me	2808 (13.1)
	<30 min	6641 (31.1)
	30 min-1 hour	6922 (32.4)
	1 hour-2 hours	3019 (14.1)
	2 hours-3 hours	964 (4.5)
	>3 hours	636 (3)
**Smoking status, n (%)**		
	Nonsmoker	13,546 (63.4)
	Former smoker	4029 (18.9)
	Current smoker	3789 (17.7)
**Job status, n (%)**		
	Decline to answer	430 (2.0)
	Do not work	8294 (38.8)
	Lost job due to COVID-19 and out of job now	1648 (7.7)
	Work from home	8366 (39.2)
	Commute to work	2626 (12.3)
**Health care–related job, n (%)**		
	No	19,589 (91.7)
	Medical student	222 (1.0)
	Volunteer/hospital management	283 (1.3)
	Nurse	305 (1.4)
	Physician	965 (4.5)
S-Anxiety^a^, median (IQR)		52 (44-60)
T-Anxiety^b^, median (IQR)		44 (39-51)

^a^S-Anxiety: State Anxiety Scale.

^b^T-Anxiety: Trait Anxiety Scale.

### Levels of Trait and State Anxiety

Scores for both T-Anxiety and S-Anxiety of survey respondents were high, with medians of 44 (IQR 39-51) and 52 (IQR 44-60), respectively.

**Figure 1:**
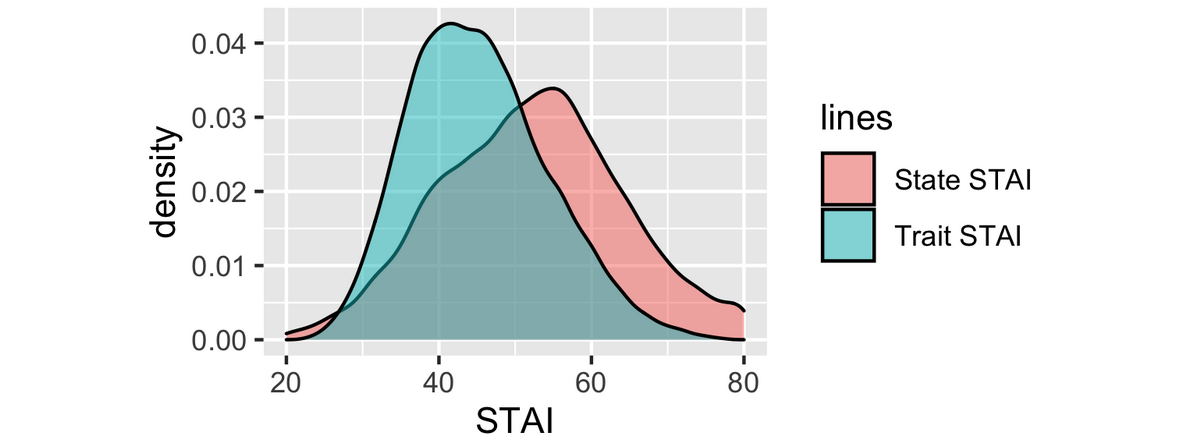
Density plot showing the difference between the state and trait anxiety based on the responses of all participants (N=21,364). STAI: State-Trait Anxiety Inventory.

**Figure 2:**
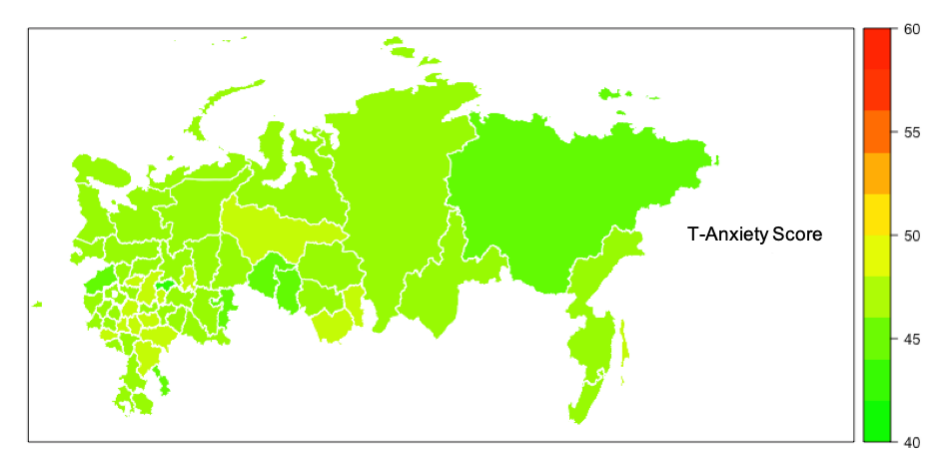
Map of Russia showing the levels of respondents’ trait anxiety (T-Anxiety). Areas with data from less than 40 respondents are not shown on the map. T-Anxiety: Trait Anxiety Scale.

**Figure 3:**
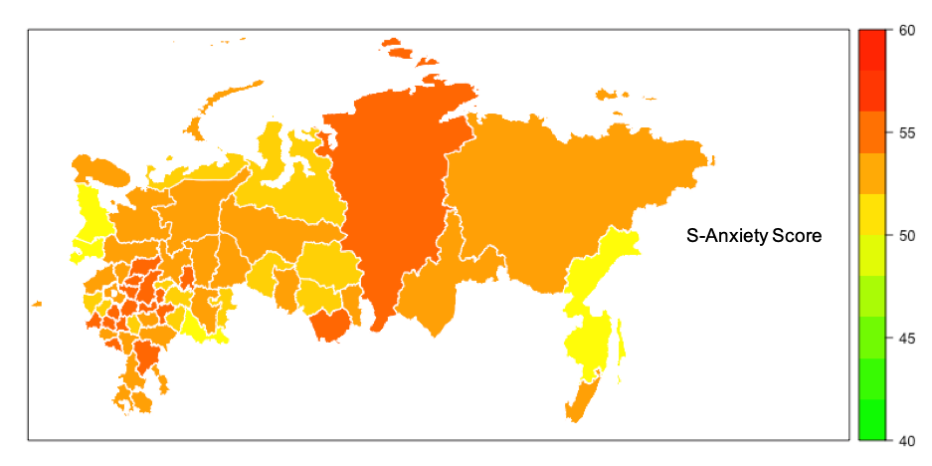
Map of Russia showing the levels of respondents’ state anxiety (S-Anxiety). Areas with data from less than 40 respondents are not shown on the map. S-Anxiety: State Anxiety Scale.

Median scores for S-Anxiety were higher than T-Anxiety across all areas of Russia (Figures 1-3 and Multimedia Appendix 1) with four areas (Belgorod, Kostroma, Mordovia, and Orel) having a score difference of 10 points or greater. In 32 out of 62 (52%) areas, the difference between S-Anxiety and T-Anxiety reached 8-9.5 points and, in 24 out of 62 (39%) areas, between 5 and 7.5 points. In two areas (Sakhalin and Karelia), the difference was less than 5 points. The difference between S-Anxiety and T-Anxiety in the largest Russian cities, Moscow and St. Petersburg, was approximately 8 points. A reference map providing detail on geographical locations of the main areas of Russia is provided in Multimedia Appendix 2.

### Determinants of State Anxiety

To assess factors associated with S-Anxiety (related to COVID-19), we developed a multiple regression model. T-Anxiety was included to adjust for pre-existing or typical state of anxiety for each individual (Table 2). The multiple logistic regression model with a cut-off value of S-Anxiety at 45 points yielded similar results (Multimedia Appendix 3).

Time spent following news on COVID-19 was significantly associated with higher scores of S-Anxiety, with one to two hours resulting in a 5.46 (95% CI 5.03 to 5.90) point difference, two to three hours in a 7.06 (95% CI 6.37 to 7.74) point difference, and more than three hours in a 8.65 (95% CI 7.82 to 9.47) point difference, all compared to up to 30 minutes per day. In addition, *job loss due to the pandemic* resulted in a 3.95 (95% CI 3.31 to 4.58) point difference, a combination of depression with either cardiovascular or respiratory conditions in a 3.19 (95% CI 1.89 to 4.49) point difference, taking neuroleptics or antidepressants in a 1.32 (95% CI 0.59 to 2.06) point difference, and smokers in a 1.16 (95% CI 0.74 to 1.50) point difference, which all had significantly higher S-Anxiety scores.

Males had a lower level of S-Anxiety than females (–4.01, 95% CI –4.45 to –3.57). Parents of children younger than 18 years and people expecting a child also had slightly lower scores of S-Anxiety, –1.44 (95% CI –1.84 to –1.04) and –1.12 (95% CI –1.79 to –0.45), respectively.

**Table 2:** Regression model assessing associations between characteristics and state anxiety scores, adjusted for the trait anxiety.

Model and variable		Coefficient	SE	*P* value	95% CI
**Sex**					
	Male vs female	–4.011	0.224	*<.001* ^a^	–4.45 to –3.572
Age		–0.021	0.011	.046	–0.042 to 0
**Marital status**					
	In relationship vs single	0.287	0.248	.25	–0.2 to 0.773
	Married vs single	0.243	0.236	.30	–0.22 to 0.706
**Have children younger than 18 years**					
	No vs yes	–1.441	0.203	*<.001*	–1.839 to –1.044
**Expecting a child**					
	No vs yes	–1.117	0.341	*.001*	–1.785 to –0.448
**Living in a capital**					
	No vs yes	–0.404	0.156	.009	–0.709 to –0.099
**Education**					
	BSc vs vocational school	0.177	0.293	.55	–0.397 to 0.751
	MSc vs vocational school	0.475	0.319	.14	–0.151 to 1.101
	Other vs vocational school	–2.016	0.951	.03	–3.88 to –0.153
	More than one degree vs vocational school	0.568	0.386	.14	–0.189 to 1.325
	Higher education in progress vs vocational school	–0.45	0.387	.25	–1.209 to 0.309
	PhD vs vocational school	–0.174	0.523	.74	–1.2 to 0.852
	School vs vocational school	–1.836	0.567	*.001*	–2.946 to –0.725
**Income (R)**					
	Decline to answer vs <20,000	–0.205	0.368	.58	–0.927 to 0.517
	20,000-35,000 vs <20,000	–0.28	0.208	.18	–0.687 to 0.128
	35,000-70,000 vs <20,000	–0.487	0.211	.02	–0.9 to –0.074
	70,000-100,000 vs <20,000	–0.311	0.274	.26	–0.847 to 0.226
	100,000-150,000 vs <20,000	–0.313	0.334	.35	–0.968 to 0.342
	>150,000 vs <20,000	–0.529	0.381	.17	–1.276 to 0.219
**Chronic medical conditions**					
	Any vs no	1.072	0.142	*<.001*	0.793 to 1.351
	Decline to answer vs no	2.774	0.58	*<.001*	1.636 to 3.911
	Depression and cardiological or respiratory vs no	3.187	0.664	*<.001*	1.885 to 4.489
	Depression or neurological vs no	0.109	0.467	.82	–0.805 to 1.024
	Food allergy/rhinitis/eczema/psoriasis vs no	0.633	0.234	.007	0.174 to 1.091
	Cardiological vs no	0.765	0.387	.048	0.007 to 1.522
	Cardiological and respiratory vs no	2.123	1.252	.09	–0.33 to 4.577
	Renal/hepatic/diabetes vs no	1.221	0.392	.002	0.452 to 1.99
	Oncology/HIV vs no	0.857	0.625	.17	–0.368 to 2.083
	Other vs no	1.281	0.165	*<.001*	0.958 to 1.605
	Respiratory vs no	0.533	0.761	.48	–0.959 to 2.025
**Medications**					
	Neuroleptics/antidepressant vs no	1.324	0.376	*<.001*	0.586 to 2.061
**Time spent on reading COVID-19 news**					
	Decline to answer vs <30 mins	2.955	1.616	.07	–0.213 to 6.122
	Do not follow vs <30 mins	–4.767	0.563	*<.001*	–5.87 to –3.663
	Do not follow but they find me vs <30 mins	1.16	0.226	*<.001*	0.716 to 1.603
	30 mins-1 hour vs <30 mins	3.083	0.173	*<.001*	2.743 to 3.423
	1-2 hours vs <30 mins	5.463	0.222	*<.001*	5.027 to 5.899
	2-3 hours vs <30 mins	7.059	0.349	*<.001*	6.374 to 7.743
	>3 hours vs <30 mins	8.645	0.421	*<.001*	7.819 to 9.471
**Smoking**					
	Former smoker vs nonsmoker	0.265	0.181	.14	–0.09 to 0.621
	Current smoker vs nonsmoker	1.115	0.193	*<.001*	0.736 to 1.494
**Job status**					
	Decline to answer vs commute to work	0.189	0.529	.72	–0.847 to 1.225
	Do not work vs commute to work	–0.443	0.242	.07	–0.916 to 0.031
	Work from home vs commute to work	–0.86	0.24	*<.001*	–1.331 to –0.39
	Lost due to COVID-19 and out of job vs commute to work	3.948	0.323	*<.001*	3.314 to 4.581
**Health care–related job**					
	Medical student vs no	–1.185	0.701	.09	–2.559 to 0.189
	Volunteer/hospital management vs no	–0.637	0.604	.29	–1.822 to 0.547
	Nurse vs no	–0.876	0.607	.15	–2.066 to 0.314
	Physician vs no	–0.886	0.349	.01	–1.57 to –0.201
T-Anxiety^b^		0.543	0.008	*<.001*	0.528 to 0.559

^a^Italics indicate significant results.

^b^T-Anxiety: Trait Anxiety Scale.

### Subgroup Analysis

An additional regression analysis in a subgroup of participants with low T-Anxiety scores was performed to see if determinants of S-Anxiety would remain the same even in people with a generally good state of calmness, confidence, and security (Multimedia Appendix 4). The effect sizes were even stronger in this group. Time spent following news on COVID-19 was found to be significantly associated with higher scores of S-Anxiety, with one to two hours resulting in an 8.06 (95% CI 4.75 to 11.38) point difference, two to three hours in a 13.77 (95% CI 7.86 to 19.67) point difference, and more than three hours in a 21.61 (95% CI 13.97 to 29.25) point difference. Job loss due to the pandemic was also strongly associated with higher S-Anxiety scores (10.95, 95% CI 6.62 to 15.28). The level of S-Anxiety for males was lower than in females (–4.011, 95% CI –4.45 to –3.57).

As most of the survey respondents were female, we ran an additional analysis to assess S-Anxiety in a subgroup of male participants (Multimedia Appendix 5), which yielded similar results to the previous regression models with respect to time spent following news on COVID-19 and job loss due to the pandemic. We investigated the influence of the survey completion date on the outcome, using S-Anxiety scores in all participants as well as the subgroup with low T-Anxiety scores (Multimedia Appendix 6) but noted no statistically significant differences.

### Confidence, Understanding, Trust, and Concerns

**Figure 4:**
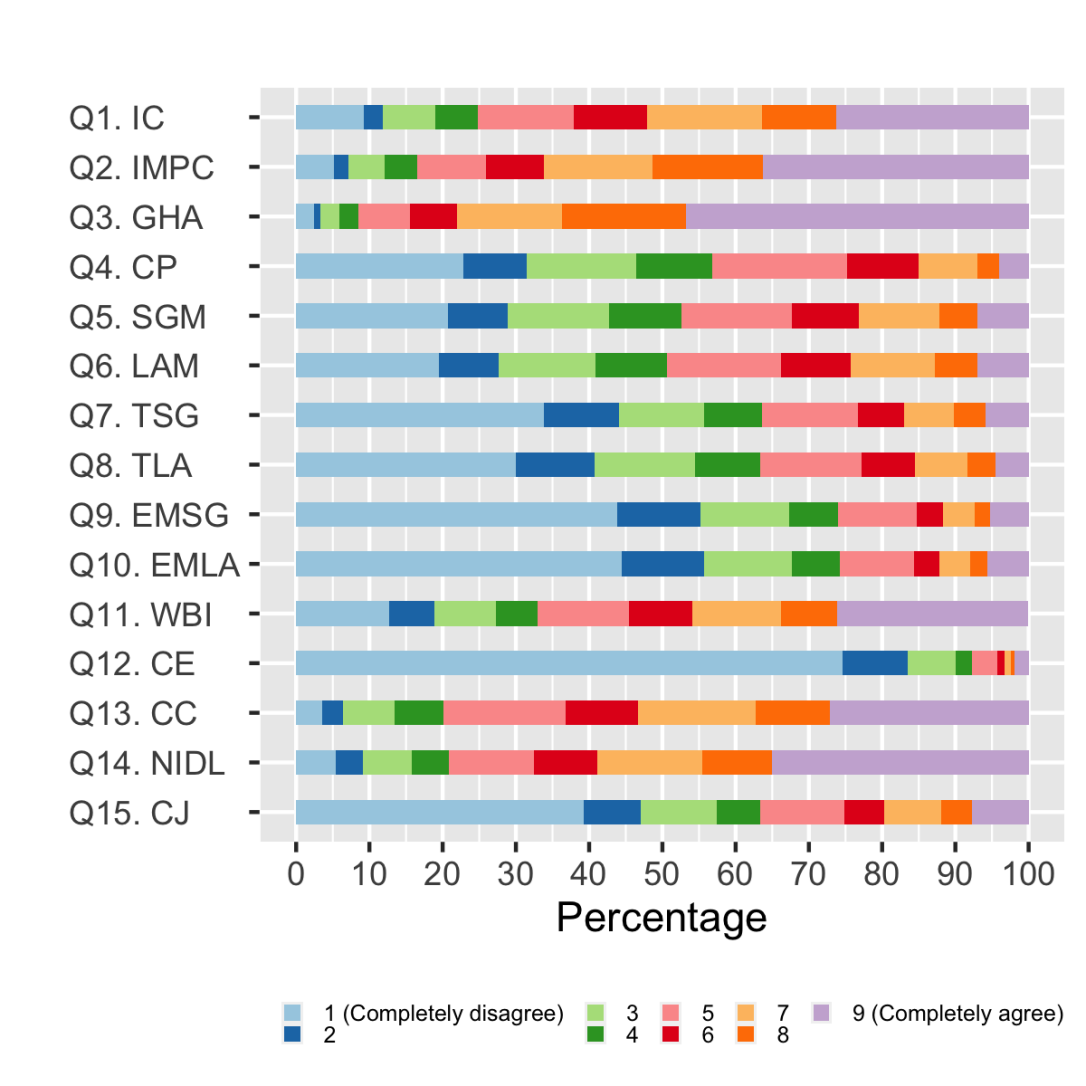
Respondents' answers to the questions addressing confidence, understanding, trust, and concerns. CC: The COVD-19 situation concerns me significantly; CE: I believe the crisis caused by COVID-19 will eventually resolve with little consequence for my country’s economy; CJ: I believe the crisis caused by COVID-19 will eventually resolve with little consequence for my job/business; CP: I think the country I am responding from is well prepared for COVID-19; EMLA: I think the measures taken by the local authorities in the city/town/village/etc against COVID-19 are excessive; EMSG: I think the measures taken by the country government against COVID-19 are excessive; GHA: I understand the guidance from health care authorities related to COVID-19; IC: I feel informed about COVID-19; IMPC: I feel informed about measures to prevent infection with COVID-19; LAM: I think that all possible local authority measures to fight COVID-19 are being taken in my city/town/village/etc; NIDL: The COVID-19 situation is negatively impacting my day-to-day life; Q: question; SGM: I think all possible government measures to fight COVID-19 are being taken in my country; TLA: I trust the local authorities in the city/town/village/etc I am responding from; TSG: I trust the government in the country I am responding from; WBI: I am worried about becoming infected with COVID-19 no matter how much I take care of myself.

Regarding questions on confidence, understanding, trust, and concerns related to COVID-19 (Figure 4 and Table 3), most of the respondents felt well-informed (scores between 7 and 9) on COVID-19 (n=11,129, 52.1%), measures to prevent infection (n=14,149, 66.2%), and understanding of the guidance from health care authorities (n=16,670, 78.0%).

**Table 3:** Respondents answers to the questions addressing confidence, understanding, trust, and concerns.^a^

Views on COVID-19		Response, median (IQR)
**Confidence in information and understanding**		
	Q^b^1. I feel informed about COVID-19.	7 (5-9)
	Q2. I feel informed about measures to prevent infection with COVID-19.	8 (5-9)
	Q3. I understand the guidance from health care authorities related to COVID-19.	8 (7-9)
**Trust to state and local authorities, and country readiness for pandemic**		
	Q4. I think the country I am responding from is well prepared for COVID-19.	4 (2-5)
	Q5. I think all possible government measures to fight COVID-19 are being taken in my country.	4 (2-6)
	Q6. I think that all possible local authority measures to fight COVID-19 are being taken in my city/town/village/etc.	4 (2-6)
	Q7. I trust the government in the country I am responding from.	3 (1-5)
	Q8. I trust the local authorities in the city/town/village/etc I am responding from.	3 (1-5)
**Governmental measures evaluation**		
	Q9. I think the measures taken by the country government against COVID-19 are excessive.	2 (1-5)
	Q10. I think the measures taken by the local authorities in the city/town/village/etc against COVID-19 are excessive.	2 (1-5)
**Worry/concern/adverse expectation**		
	Q11. I am worried about becoming infected with COVID-19 no matter how much I take care of myself.	6 (3-9)
	Q12. I believe the crisis caused by COVID-19 will eventually resolve with little consequence for my country’s economy.	1 (1-2)
	Q13. The COVD-19 situation concerns me significantly.	7 (5-9)
	Q14. The COVID-19 situation is negatively impacting my day-to-day life.	7 (5-9)
	Q15. I believe the crisis caused by COVID-19 will eventually resolve with little consequence for my job/business.	3 (1-6)

^a^Answers were provided with a 9-point Likert scale, where 1 is *completely disagree* and 9 is *completely agree*.

^b^Q: question.

Out of the 21,364 participants, very few people considered that the country was well-prepared for the pandemic (n=3202, 15.0%) and that all possible state and local government measures to fight COVID-19 were being taken (n=4950, 23% and n=5189, 24%, respectively), and more than half of the respondents reported low trust in the state government and local authorities (n=11,890, 56% and n=11,624, 54%, respectively). Interestingly, most participants did not consider the measures taken by the state and local government to be excessive (n=14,382, 67% and n=14,459, 68%, respectively). Regarding the economic ramifications, 19,240 (90%) respondents did not believe that the crisis caused by COVID-19 will eventually resolve with little consequence for the country’s economy, and 12,269 (57%) were worried about the consequences to their business or employment. More than half of participants (n=12,570, 58.8%) reported the pandemic was “negatively impacting my day to day life” and were extremely concerned with the situation (11,389, 53.3%), and 9812 (46%) were worried about becoming infected “no matter how much I take care of myself.”

**Figure 5:**
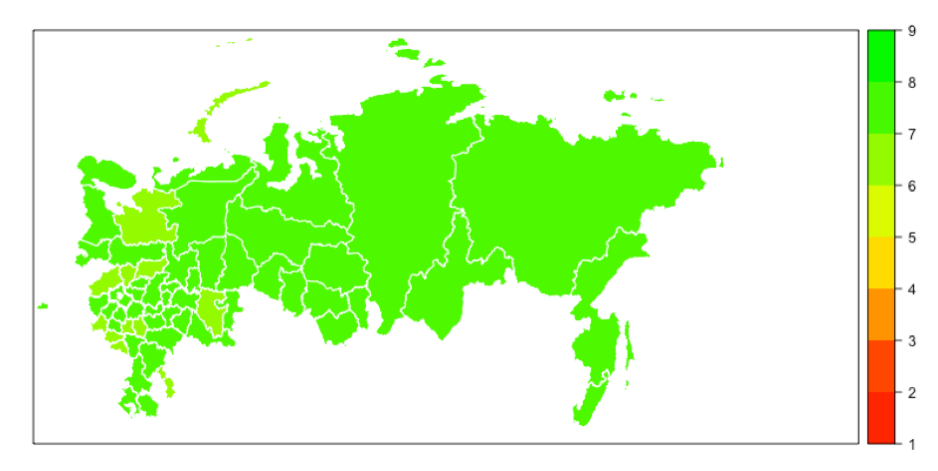
Map of Russia showing the levels of respondents’ confidence in information and understanding. Areas with data from less than 40 respondents are not shown on the map. The combined median score on confidence in information and understanding was used (Question [Q]1. I feel informed about COVID-19; Q2. I feel informed about measures to prevent infection with COVID-19; Q3. I understand the guidance from health care authorities related to COVID-19). Respondents were provided with a 9-point Likert scale, where 1 is completely disagree and 9 is completely agree.

Respondents demonstrated good confidence in information and in understanding of COVID-19 across most areas of Russia (Multimedia Appendix 7 and Figure 5), with 51 out of 62 (82%) areas having combined median scores of seven or more. The rest of the country had a median score between 6 and 7.

**Figure 6:**
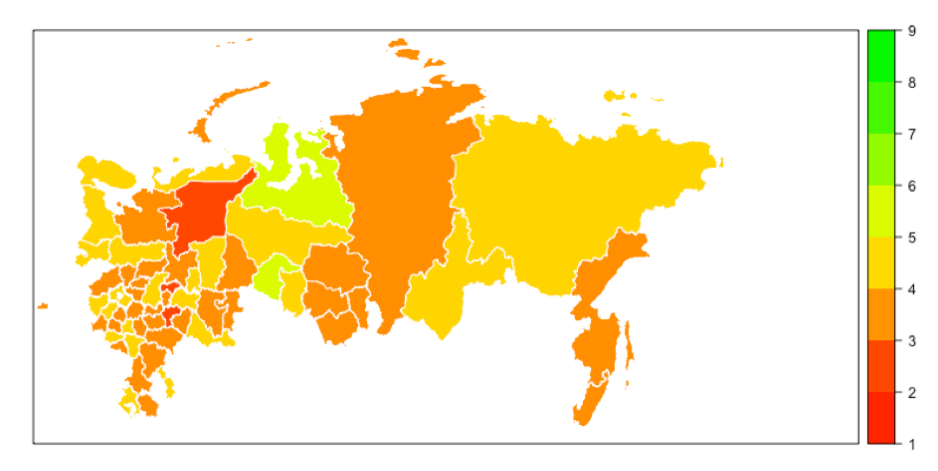
Map of Russia showing the levels of respondents’ trust to state and local authorities, and country readiness for the pandemic. Areas with data from less than 40 respondents are not shown on the map. The combined median score on trust to state and local authorities, and country readiness for the pandemic (Question [Q]4. I think the country I am responding from is well prepared for COVID-19; Q5. I think all possible government measures to fight COVID-19 are being taken in my country; Q6. I think that all possible local authority measures to fight COVID-19 are being taken in my city/town/village/etc; Q7. I trust the government in the country I am responding from; Q8. I trust the local authorities in the city/town/village/etc I am responding from). Respondents were provided with a 9-point Likert scale, where 1 is completely disagree and 9 is completely agree.

Combined median scores on trust of state and local authorities, and country readiness for the pandemic did not exceed 5 on a nine-point scale across Russia, reaching 5 in two areas only (Tyumen’ and Yamal-Nenetsk). In 23 out of 62 (37%) areas, the median score varied between 4 and 4.8; in 33 out of 62 (53%) areas, the median score ranged between 3 and 3.9. A median score below 3 was recorded in three areas of Russia (Komi, Mari El, and Ul’yanovsk). The median score in Moscow was 4 points, with an even lower median (3.1) seen in respondents from St. Petersburg. Figure 6 shows median scores in areas across Russia.

## Discussion

### Principal Findings

The findings herein demonstrate higher state versus trait anxiety among a large sample of people residing in Russia during the COVID-19 pandemic. State anxiety was strongly associated with the amount of time spent following news of COVID-19, as well as job loss during the pandemic. Although our study design and analysis did not allow us to assess causal inference, the amount of time spent following news may be driven in part by low levels of trust in state and local authorities.

Widely accepted reference norms for STAI in the Russian population suggest that scores up to 30 are equivalent to a low level of anxiety, while scores above 45 indicate a high or *clinical* level of anxiety. The median scores for S-Anxiety in our online survey respondents were exceedingly high (52, IQR 44-60), which was expected, but this level of anxiety in a large sample size of young adults is concerning.

Some of the findings may be mediated by the timing of survey administration in conjunction with recent governmental actions taken less than a week prior, when the Russian President Vladimir Putin announced the prolongation of “the official non-work period” until the end of April and alluded to regional decision making regarding public health measures [7]. By that time, several (but not all) regions including Moscow had introduced social or physical distancing measures, the stringency of which were further adjusted over the course of this study while remaining variable across the country. Therefore, the results must be viewed through a lens of a fluid and evolving situation in a geographically large and culturally diverse country with variable infection rates and public health needs when analyzing interregional variation of the anxiety state. Furthermore, no data regarding anxiety, trust, or other data reported herein were available to help inform potential decisions. Such data are now available and support interventions for a psychologically burdened people [16]. Electronic mental health interventions such as *CoPE It* could be made available as evidence-based psychotherapeutic and psychological support to overcome psychological distress [17].

Moreover, similarly to other countries, additional support measures for the private sector and guarantees of wage retention for the workers of nongovernmental organizations have evolved since the launch of this survey. A window existed where respondents were surveyed between announcements, which may have influenced perception of the adequacy of the response, in particular with oil price fluctuations occurring just prior to the fielding of this survey [18]. This lack of hindsight clarity at the beginning stages could explain the observed anxiety related to job loss and the lack of belief in the crisis caused by COVID-19 resolving with little consequence for the respondents’ jobs and for the country’s economy. This is why longitudinal assessment of this population is important, to show if there is resolution of some of the trends.

Previous research suggested that high frequency of risk-elevating messages in the news may contribute to increased concerns of the public in relation to infectious diseases, as it was witnessed with the Ebola virus disease in the US population [19]. Our findings are in agreement with previous data both during the current pandemic [20,21] as well as the severe acute respiratory syndrome outbreak in 2006 [22]; the time of media consumption was a major factor associated with higher S-Anxiety in our respondents. The effect was particularly strong among participants with low T-Anxiety, which could indicate ceiling effects among those with high T-Anxiety (ie, individuals with high T-Anxiety already have increased anxiety, which does not leave enough space for S-Anxiety to rise to a large extent). Another interpretation is that subjects who usually having low anxiety in normal life show a stronger response during the stressful time of a pandemic. Individuals with higher T-Anxiety might be more prone to increased duration or frequency of media consumption; however, a strong association among those with low T-Anxiety may point toward the directionality of media consumption causing S-Anxiety rather than the other way around, which is supported by previously published data [23]. The design of this study does not allow establishment of causality, but this hypothesis may merit further exploration in future research.

Our data support the findings by Ni et al [20], suggesting that spending ≥2 hours a day following COVID-19–related news was associated with probable depression and anxiety in adults. Social media sources may impose a danger to mental health during lockdown due to the high volume of contradictory information they may deliver [22,24,25], which is particularly evident in light of quarantine measures imposed. A recent report by Tangcharoensathien et al [26] highlighted the importance of interaction with social media platforms to provide reliable information and keep the infodemic under control, underlining the necessity of keeping closer attention to the coherence of information in the media and to take specific action to alleviate their impact on mental health.

The impact of the COVID-19 pandemic on the Russian population remains largely unknown with a limited number of studies on a small sample size available [27-29]. Sorokin et al [27] reported an overall moderate level of anxiety and found similar associations between unemployment, female gender, and lower education with the level of distress. Similarly, high levels of anxiety were found in the Russian student population [28,29], but due to the difference in scales used, the results cannot be compared with our data.

### Strengths and Limitations

The size of the data set, assessing mental aspects of a sizeable portion of the Russian population during the pandemic, is a defined strength of the study. The survey resulted in a reputable completion rate compared with an average response rate in online surveys (33%) [30]. This may be a reflection of social or physical isolation and policies urging people to shelter-in-place, who could then dedicate more time to responding.

There were several limitations to the study. Several items were not standardized and validated, although we were careful to include others that were. However, a broad international multidisciplinary team of researchers, with expertise in psychology, epidemiology, and clinical medicine including infectious diseases were involved in this ad hoc survey development to measure the pandemic response. The survey was distributed online, with the help of influencers via social networks, media, and search engines. This may have introduced selection bias due to the probability that people using media websites and persons who follow online influencers and take a questionnaire may possess higher health literacy and be more informed on a wide range of topics, which reduces generalizability of the findings to the average Russian citizen. However, Russia is among the top-10 countries in the world with the highest number of internet users [31], and most of the people in the studied age group use internet on a regular basis all over Russia. Another concern was age bias because many of the respondents were young adults, and the views of people 65 years and older, the most vulnerable population during the COVID-19 pandemic, remained uncaptured. Finally, another limitation of this study is the disproportionately large number of female respondents, which is, however, a common observation in online and paper-based surveys [32,33]. The gender difference is likely to be secondary to the means of the questionnaire’s distribution, that is, via social media and influencers.

### Conclusions

The results of this survey suggest a higher rate of S-Anxiety when compared with T-Anxiety diffusely among the Russian population. Media consumption, job loss, and associated uncertainty around future employment prospects due to the pandemic were strongly associated with increased S-Anxiety. Given the evolving pandemic situation, further research is needed to track the trajectory of perceptions regarding trust and the perception of the adequacy of the governmental response. This also provides time for some of the information contained herein to help inform policy and direct intervention at a segment of the population at high risk for mental health issues related to the pandemic and what influences the anxiety. It is important to address the COVID-19 pandemic and the *infodemic* in tandem by understanding if mass communication impacts state anxiety. Our findings will increase our understanding of the risks and consequences of social isolation on the population and how these are informed by rapidly changing data, an endless news cycle, and social media.

In their position paper, Holmes et al [5], as well as the WHO experts [6], called for immediate action to assess “the effect of repeated media consumption about COVID-19 in traditional and social media on mental health” and “the role of repeated media consumption in amplifying distress and anxiety” [5]. Our data confirm the association between an excessive reception of information on COVID-19 through the media and S-Anxiety. Lack of trust in the state and local authorities is also worrying. Governments must take note and consider how presentation might be adjusted to avoid excess anxiety in the population.

## Appendix

Multimedia Appendix 1 Median scores of trait (State-Trait Anxiety Inventory–trait) and state (State-Trait Anxiety Inventory–state) anxiety reported by the respondents, residing in different regions (with 40 respondents or more) of the Russian Federation.

Multimedia Appendix 2 Reference map of the Russian Federation.

Multimedia Appendix 3 Multiple logistic regression model assessing associations between characteristics and state anxiety scores, adjusted for the trait anxiety. Statistically significant results presented in bold.

Multimedia Appendix 4 Regression model assessing associations between characteristics and state anxiety scores in a subset of respondents with low trait anxiety scores (n=683). Statistically significant results presented in bold.

Multimedia Appendix 5 Regression model assessing associations between characteristics and state anxiety scores in a subset of respondents with low trait anxiety scores (n=683). Statistically significant results presented in bold.

Multimedia Appendix 6 Changes in State Anxiety Scale scores over time in (a) all respondents and (b) a subset of respondents with low trait anxiety scores.

Multimedia Appendix 7 Median scores reported by the respondents residing in different regions (with 40 respondents or more) of Russian Federation with regards to (a) confidence in information and understanding, combined median score on confidence in information and understanding was used (Question [Q]1. I feel informed about COVID-19; Q2. I feel informed about measures to prevent infection with COVID-19; Q3. I understand the guidance from health care authorities related to COVID-19) and (b) combined median score on trust to state and local authorities, and country readiness for pandemic (Q4. I think the country I am responding from is well prepared for COVID-19; Q5. I think all possible government measures to fight COVID-19 are being taken in my country; Q6. I think that all possible local authority measures to fight COVID-19 are being taken in my city/town/village/etc; Q7. I trust the government in the country I am responding from; Q8. I trust the local authorities in the city/town/village/etc I am responding from). Respondents were provided with a 9-point Likert scale, where 1 is completely disagree and 9 is completely agree.

Multimedia Appendix 8 Regression model assessing associations between characteristics and state anxiety, including trust to state and local authorities, country readiness for the pandemic, and confidence in information and understanding. Statistically significant results presented in bold.

## Abbreviations

CHERRIES: Checklist for Reporting Results of Internet E-Surveys
STAI: State-Trait Anxiety Inventory
S-Anxiety: State Anxiety Scale
T-Anxiety: Trait Anxiety Scale
WHO: World Health Organization
