# Supplementary material for: Excessive Media Consumption About COVID-19 is Associated With Increased State Anxiety: Outcomes of a Large Online Survey in Russia
Source: J Med Internet Res. 2020 Sep 11;22(9):e20955. doi: 10.2196/20955 (PMC7490003; doi:10.2196/20955)
Supplement: Multimedia Appendix 1 [file jmir_v22i9e20955_app1.docx]

**Table S1** Median scores of trait (STAI-T) and state (STAI-S) anxiety reported by the respondents, residing in different regions (with 40 respondents or more) of Russian Federation.

| **Area** | **T-Anxiety** | **S-Anxiety** | **Difference between**  **S-Anxiety and T-Anxiety** |
| --- | --- | --- | --- |
| **Altay** | 46 | 54 | 8 |
| **Arkhangel'sk** | 44.5 | 53 | 8.5 |
| **Astrakhan'** | 43.5 | 53 | 9.5 |
| **Bashkortostan** | 45 | 53 | 8 |
| **Belgorod** | 44.5 | 55 | 10.5 |
| **Bryansk** | 45 | 54 | 9 |
| **Chelyabinsk** | 43 | 50 | 7 |
| **Chuvash** | 46 | 54.5 | 8.5 |
| **City of St. Petersburg** | 45 | 53 | 8 |
| **Irkutsk** | 45 | 52 | 7 |
| **Ivanovo** | 47 | 54 | 7 |
| **Kaliningrad** | 44 | 53 | 9 |
| **Kaluga** | 45 | 50 | 5 |
| **Karelia** | 45 | 49.5 | 4.5 |
| **Kemerovo** | 47 | 52 | 5 |
| **Khabarovsk** | 44 | 49 | 5 |
| **Khanty-Mansiysk** | 46 | 53 | 7 |
| **Kirov** | 45 | 53 | 8 |
| **Komi** | 45 | 53.5 | 8.5 |
| **Kostroma** | 45 | 55 | 10 |
| **Krasnodar** | 45 | 53 | 8 |
| **Krasnoyarsk** | 45 | 54 | 9 |
| **Kursk** | 46.5 | 53.5 | 7 |
| **Leningrad Oblast’** | 44 | 49 | 5 |
| **Lipetsk** | 47.5 | 55 | 7.5 |
| **Mariy-El** | 41 | 50 | 9 |
| **Mordovia** | 45 | 55 | 10 |
| **Moscow City** | 44 | 52 | 8 |
| **Moskva** | 45 | 53 | 8 |
| **Murmansk** | 44 | 53 | 9 |
| **Nenets** | 45 | 51 | 6 |
| **Nizhniy Novgorod** | 46 | 55 | 9 |
| **Novosibirsk** | 44 | 50 | 6 |
| **Omsk** | 43.5 | 52.5 | 9 |
| **Orel** | 44 | 54 | 10 |
| **Orenburg** | 44.5 | 49.5 | 5 |
| **Penza** | 46 | 53 | 7 |
| **Perm'** | 44 | 52 | 8 |
| **Primor'ye** | 44 | 53 | 9 |
| **Rostov** | 44 | 53 | 9 |
| **Ryazan'** | 46 | 55 | 9 |
| **Sakha** | 43 | 52 | 9 |
| **Sakhalin** | 46 | 49.5 | 3.5 |
| **Samara** | 44 | 51 | 7 |
| **Saratov** | 46 | 52 | 6 |
| **Smolensk** | 44.5 | 51 | 6.5 |
| **Stavropol'** | 45 | 52 | 7 |
| **Sverdlovsk** | 44 | 53 | 9 |
| **Tambov** | 46.5 | 51.5 | 5 |
| **Tatarstan** | 45 | 51 | 6 |
| **Tomsk** | 45 | 51 | 6 |
| **Tula** | 45 | 54 | 9 |
| **Tver'** | 43.5 | 52.5 | 9 |
| **Tyumen'** | 42 | 50 | 8 |
| **Udmurt** | 47 | 54 | 7 |
| **Ul'yanovsk** | 45 | 54 | 9 |
| **Vladimir** | 45 | 54.5 | 9.5 |
| **Volgograd** | 46 | 54 | 8 |
| **Vologda** | 45 | 52.5 | 7.5 |
| **Voronezh** | 44 | 52 | 8 |
| **Yamal-Nenets** | 45 | 51 | 6 |
| **Yaroslavl'** | 44 | 52 | 8 |

**Table S1** Median scores of trait (STAI-T) and state (STAI-S) anxiety reported by the respondents, residing in different regions (with 40 respondents or more) of Russian Federation.
