# Supplementary figures and images for: Excessive Media Consumption About COVID-19 is Associated With Increased State Anxiety: Outcomes of a Large Online Survey in Russia
Source: J Med Internet Res. 2020 Sep 11;22(9):e20955. doi: 10.2196/20955 (PMC7490003; doi:10.2196/20955)

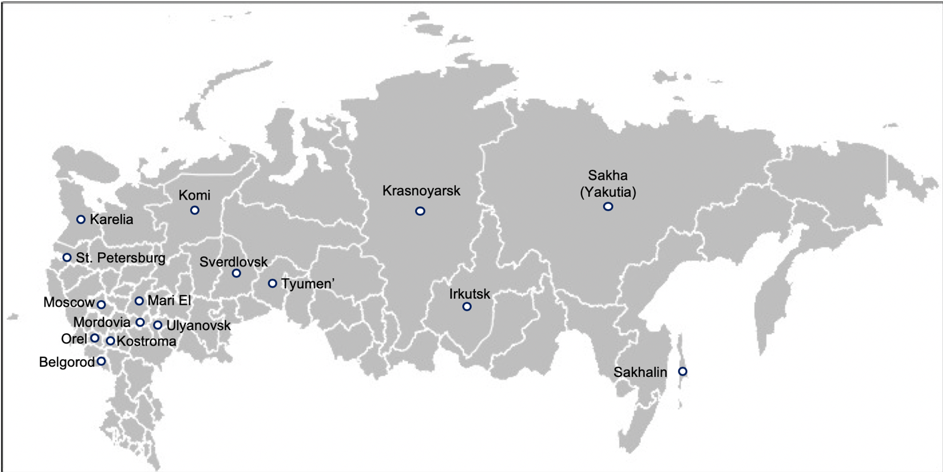

Supplement: Multimedia Appendix 2 [file jmir_v22i9e20955_app2.png]

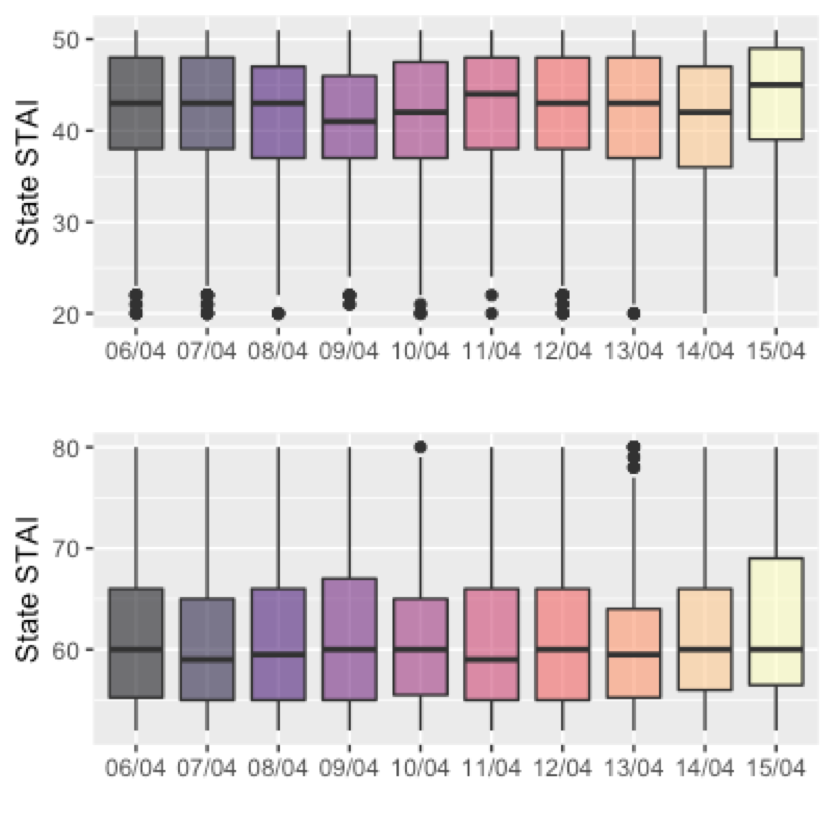

Supplement: Multimedia Appendix 6 [file jmir_v22i9e20955_app6.png]
