# Supplementary material for: Excessive Media Consumption About COVID-19 is Associated With Increased State Anxiety: Outcomes of a Large Online Survey in Russia
Source: J Med Internet Res. 2020 Sep 11;22(9):e20955. doi: 10.2196/20955 (PMC7490003; doi:10.2196/20955)
Supplement: Multimedia Appendix 3 [file jmir_v22i9e20955_app3.docx]

**Table 2** Multiple logistic regression model assessing associations between characteristics and state anxiety scores. adjusted for the trait anxiety.

Statistically significant results presented in bold.

| **Model and variable** | **OR** | **Std. Err** | **P value** | **95% CI** |
| --- | --- | --- | --- | --- |
| **Sex** |  |  |  |  |
| Male vs Female | 0.490 | 0.051 | **<.001** | 0.443  to  0.542 |
| **Age** | 0.999 | 0.003 | .629 | 0.994  to  1.004 |
| **Marital Status** |  |  |  |  |
| In relationship vs Single | 1.093 | 0.06 | .138 | 0.972  to  1.229 |
| Married vs Single | 1.139 | 0.057 | .022 | 1.019  to  1.272 |
| **Have children below the age of 18** |  |  |  |  |
| No vs Yes | 0.858 | 0.049 | .002 | 0.78  to  0.944 |
| **Expecting a child** |  |  |  |  |
| No vs Yes | 0.818 | 0.082 | .014 | 0.696  to  0.959 |
| **Living in a capital** |  |  |  |  |
| No vs Yes | 0.909 | 0.038 | .012 | 0.845  to  0.979 |
| **Education** |  |  |  |  |
| BSc vs Vocational school | 1.114 | 0.072 | .136 | 0.966  to  1.282 |
| MSc vs Vocational school | 1.106 | 0.078 | .197 | 0.948  to  1.289 |
| Other vs Vocational school | 0.914 | 0.229 | .694 | 0.587  to  1.446 |
| More than one degree vs Vocational school | 1.089 | 0.093 | .364 | 0.906  to  1.307 |
| Higher education in progress vs Vocational school | 1.001 | 0.095 | .993 | 0.831  to  1.205 |
| PhD vs Vocational school | 1.021 | 0.124 | .866 | 0.802  to  1.301 |
| School vs Vocational school | 0.920 | 0.14 | .555 | 0.701  to  1.213 |
| **Income (RUB)** |  |  |  |  |
| Decline to answer vs <20,000 | 0.900 | 0.089 | .235 | 0.757  to  1.072 |
| 20,000-35.000 vs <20.000 | 0.927 | 0.051 | .139 | 0.838  to  1.025 |
| 35,000-70,000 vs <20,000 | 0.900 | 0.052 | .042 | 0.813  to  0.996 |
| 70,000-100.000 vs <20,000 | 0.951 | 0.066 | .45 | 0.835  to  1.083 |
| 100,000-150,000 vs <20.000 | 0.904 | 0.08 | .205 | 0.773  to  1.057 |
| >150,000 vs <20,000 | 0.893 | 0.09 | .205 | 0.749  to  1.065 |
| **Chronic medical conditions** |  |  |  |  |
|  |  |  |  |  |
| Any vs No | 1,195 | 0,034 | **<.001** | 1.118  to  1.278 |
| Decline to answer vs No | 1.982 | 0.159 | **<.001** | 1.459  to  2.729 |
| Depression and (Cardiological or Respiratory) vs No | 1.493 | 0.19 | .035 | 1.038  to  2.191 |
| Depression or Neurological vs No | 1.027 | 0.123 | .828 | 0.809  to  1.313 |
| FoodAllergy/Rhinitis/Eczema/Psorias vs No | 1.131 | 0.055 | .027 | 1.015  to  1.26 |
| Cardiological vs No | 1.022 | 0.091 | .806 | 0.856  to  1.224 |
| Cardiological and Respiratory vs No | 1.376 | 0.314 | .309 | 0.759  to  2.615 |
| Renal/Hepatic/Diabetes vs No | 1.183 | 0.096 | .081 | 0.981  to  1.432 |
| Oncology/HIV vs No | 1.115 | 0.153 | .476 | 0.83  to  1.509 |
| Other vs No | 1.265 | 0.04 | **<.001** | 1.169  to  1.369 |
| Respiratory vs No | 0.931 | 0.186 | 0.7 | 0.648  to  1.348 |
| **Medications** |  |  |  |  |
| Neuroleptics/Antidepressant vs No | 1.100 | 0.104 | .362 | 0.898  to  1.353 |
| **Time spent on reading Covid news** |  |  |  |  |
| Decline to answer vs <30 mins | 1.559 | 0.387 | .251 | 0.749  to  3.454 |
| Do not follow vs <30 mins | 0.379 | 0.128 | **<.001** | 0.295  to  0.487 |
| Do not follow but they find me vs <30 mins | 1.088 | 0.051 | .105 | 0.983  to  1.203 |
| 30min-1h vs <30 mins | 1.650 | 0.041 | **<.001** | 1.524  to  1.787 |
| 1-2h vs <30 mins | 2.494 | 0.057 | **<.001** | 2.231  to  2.795 |
| 2-3h vs <30 mins | 3.130 | 0.101 | **<.001** | 2.575  to  3.831 |
| 3h+ vs <30 mins | 3.931 | 0.134 | **<.001** | 3.043  to  5.139 |
| **Smoking** |  |  |  |  |
| Former smoker vs Non-smoker | 1.005 | 0.044 | .909 | 0.923  to  1.095 |
| Current smoker vs Non-smoker | 1.223 | 0.048 | **<.001** | 1.114  to  1.342 |
| **Job Status** |  |  |  |  |
| Decline to answer vs Commute to work | 1.119 | 0.132 | .397 | 0.865  to  1.455 |
| Do not work vs Commute to work | 0.914 | 0.058 | .121 | 0.815  to  1.024 |
| Work from home vs Commute to work | 0.862 | 0.058 | .01 | 0.77  to  0.965 |
| Lost due to Covid and out of job vs Commute to work | 2.008 | 0.085 | **<.001** | 1.701  to  2.372 |
| **Healthcare-related job** |  |  |  |  |
| Medical student vs No | 0.686 | 0.165 | .022 | 0.498  to  0.951 |
| Volunteer/Hospital Management vs No | 0.874 | 0.147 | .357 | 0.657  to  1.169 |
| Nurse vs No | 0.688 | 0.146 | .01 | 0.518  to  0.919 |
| Physician vs No | 0.892 | 0.083 | .17 | 0.758  to  1.051 |
| **T-Anxiety** | 1.107 | 0.002 | **<.001** | 1.102  to  1.112 |
