# Supplementary material for: Excessive Media Consumption About COVID-19 is Associated With Increased State Anxiety: Outcomes of a Large Online Survey in Russia
Source: J Med Internet Res. 2020 Sep 11;22(9):e20955. doi: 10.2196/20955 (PMC7490003; doi:10.2196/20955)
Supplement: Multimedia Appendix 4 [file jmir_v22i9e20955_app4.docx]

**Table S3** Regression model assessing associations between characteristics and state anxiety scores in a subset of respondents with low trait anxiety scores (n=683).

Statistically significant results presented in bold.

| **Model and variable** | **Coef.** | **Std. Err** | **P value** | **95% CI** |
| --- | --- | --- | --- | --- |
| **Sex** |  |  |  |  |
| Male vs Female | -7.371 | 1.301 | ***P*<.001** | -9.926 to -4.816 |
| **Age** | -0.167 | 0.072 | *P*=.022 | -0.309 to -0.025 |
| **Marital Status** |  |  |  |  |
| In relationship vs Single | -0.167 | 1.68 | *P*=.921 | -3.465 to 3.132 |
| Married vs Single | -0.388 | 1.523 | *P*=.799 | -3.38 to 2.603 |
| **Have children below the age of 18** |  |  |  |  |
| No vs Yes | -2.018 | 1.336 | *P*=.131 | -4.642 to 0.606 |
| **Expecting a child** |  |  |  |  |
| No vs Yes | -1.963 | 2.316 | *P*=.397 | -6.511 to 2.584 |
| **Living in a capital** |  |  |  |  |
| No vs Yes | -1.071 | 1.09 | *P*=.326 | -3.212 to 1.07 |
| **Education** |  |  |  |  |
| BSc vs Vocational school | -1.898 | 2.547 | *P*=.456 | -6.9 to 3.104 |
| MSc vs Vocational school | -0.504 | 2.65 | *P*=.849 | -5.707 to 4.698 |
| Other vs Vocational school |  |  |  |  |
| More than one degree vs Vocational school | -1.419 | 2.915 | *P*=.626 | -7.143 to 4.304 |
| Higher education in progress vs Vocational school | -3.946 | 2.998 | *P*=.189 | -9.833 to 1.941 |
| PhD vs Vocational school | -0.463 | 3.584 | *P*=.897 | -7.502 to 6.575 |
| School vs Vocational school | -5.874 | 3.907 | *P*=.133 | -13.547 to 1.799 |
| **Income (RUB)** |  |  |  |  |
| Decline to answer vs <20,000 | 4.676 | 2.609 | *P*=.074 | -0.446 to 9.799 |
| 20,000-35,000 vs <20,000 | 2.631 | 1.787 | *P*=.141 | -0.878 to 6.14 |
| 35,000-70,000 vs <20,000 | 1.446 | 1.686 | *P*=.391 | -1.865 to 4.757 |
| 70,000-100,000 vs <20,000 | 1.132 | 1.913 | *P*=.554 | -2.624 to 4.888 |
| 100,000-150,000 vs <20,000 | -0.38 | 2.255 | *P*=.866 | -4.808 to 4.048 |
| 150,000+ vs <20,000 | 1.167 | 2.253 | *P*=.605 | -3.256 to 5.59 |
| **Chronic medical conditions** |  |  |  |  |
| Decline to answer vs No | 12.342 | 5.46 | *P*=.024 | 1.621 to 23.064 |
| Depression and (Cardiological or Respiratory) vs No | -0.368 | 5.492 | *P*=.947 | -11.152 to 10.416 |
| Depression or Neurological vs No | 0.702 | 7.533 | *P*=.926 | -14.09 to 15.495 |
| FoodAllergy/Rhinitis/Eczema/Psorias vs No | 0.062 | 1.561 | *P*=.969 | -3.003 to 3.126 |
| Cardiological vs No | -3.952 | 2.916 | *P*=.176 | -9.678 to 1.775 |
| Cardiological and Respiratory vs No | -2.549 | 12.488 | *P*=.838 | -27.072 to 21.974 |
| Renal/Hepatic/Diabetes vs No | 0.555 | 3.018 | *P*=.854 | -5.372 to 6.482 |
| Oncology/HIV vs No | -1.35 | 4.253 | *P*=.751 | -9.701 to 7.002 |
| Other vs No | 1.64 | 1.184 | *P*=.166 | -0.685 to 3.964 |
| Respiratory vs No | 12.703 | 5.364 | *P*=.018 | 2.17 to 23.236 |
| **Medications** |  |  |  |  |
| Neuroleptics/Antidepressant vs No | 8.946 | 4.573 | *P*=.051 | -0.033 to 17.926 |
| **Time spent on reading Covid news** |  |  |  |  |
| Decline to answer vs <30 mins | -10.643 | 9.401 | *P*=.258 | -29.103 to 7.818 |
| Do not follow vs <30 mins | -7.288 | 2.593 | *P*=.005 | -12.38 to -2.196 |
| Do not follow but they find me vs <30 mins | 2.935 | 1.398 | *P*=.036 | 0.19 to 5.68 |
| 30min-1h vs <30 mins | 6.306 | 1.232 | ***P*<.001** | 3.888 to 8.725 |
| 1-2h vs <30 mins | 8.064 | 1.69 | ***P*<.001** | 4.746 to 11.381 |
| 2-3h vs <30 mins | 13.765 | 3.008 | ***P*<.001** | 7.859 to 19.672 |
| 3h+ vs <30 mins | 21.611 | 3.89 | ***P*<.001** | 13.972 to 29.249 |
| **Smoking** |  |  |  |  |
| Former smoker vs Non-smoker | 0.397 | 1.426 | 0.781 | -2.404 to 3.198 |
| Current smoker vs Non-smoker | 0.668 | 1.271 | 0.6 | -1.827 to 3.162 |
| **Job Status** |  |  |  |  |
| Decline to answer vs Commute to work | 0.091 | 3.845 | 0.981 | -7.459 to 7.64 |
| Do not work vs Commute to work | -0.702 | 1.795 | 0.696 | -4.226 to 2.822 |
| Work from home vs Commute to work | 2.093 | 1.654 | 0.206 | -1.155 to 5.341 |
| Lost due to Covid and out of job vs Commute to work | 10.947 | 2.206 | **<0.001** | 6.616 to 15.278 |
| **Healthcare-related job** |  |  |  |  |
| Medical student vs No | -5.074 | 5.754 | 0.378 | -16.373 to 6.226 |
| Volunteer/Hospital Management vs No | -3.773 | 4.48 | 0.4 | -12.57 to 5.024 |
| Nurse vs No | 1.188 | 7.368 | 0.872 | -13.28 to 15.656 |
| Physician vs No | 2.055 | 2.261 | 0.364 | -2.385 to 6.494 |
