# Supplementary material for: Excessive Media Consumption About COVID-19 is Associated With Increased State Anxiety: Outcomes of a Large Online Survey in Russia
Source: J Med Internet Res. 2020 Sep 11;22(9):e20955. doi: 10.2196/20955 (PMC7490003; doi:10.2196/20955)
Supplement: Multimedia Appendix 5 [file jmir_v22i9e20955_app5.docx]

**Table S4** Regression model assessing associations between characteristics and state anxiety for a subset of male respondents (n = 2755).

Statistically significant results presented in bold.

| **Model and variable** | **Coef.** | **Std. Err** | **P value** | **95% CI** |
| --- | --- | --- | --- | --- |
| **Age** | 0.008 | 0.025 | *P*=.747 | -0.041 to 0.057 |
| **Marital Status** |  |  |  |  |
| In relationship vs Single | -0.572 | 0.482 | *P*=.236 | -1.516 to 0.373 |
| Married vs Single | 0.544 | 0.54 | *P*=.314 | -0.514 to 1.603 |
| **Have children below the age of 18** |  |  |  |  |
| No vs Yes | -0.429 | 0.515 | *P*=.405 | -1.439 to 0.581 |
| **Expecting a child** |  |  |  |  |
| No vs Yes | 0.318 | 1.191 | *P*=.789 | -2.018 to 2.654 |
| **Living in a capital** |  |  |  |  |
| No vs Yes | -0.89 | 0.405 | *P*=.028 | -1.683 to -0.096 |
| **Education** |  |  |  |  |
| BSc vs Vocational school | -0.175 | 0.504 | *P*=.728 | -1.162 to 0.812 |
| MSc vs Vocational school | -4.224 | 3.062 | *P*=.168 | -10.228 to 1.779 |
| Other vs Vocational school | -1.239 | 0.895 | *P*=.166 | -2.995 to 0.516 |
| More than one degree vs Vocational school | -0.539 | 0.606 | *P*=.374 | -1.726 to 0.649 |
| Higher education in progress vs Vocational school | 0.693 | 0.733 | *P*=.344 | -0.744 to 2.13 |
| PhD vs Vocational school | -1.019 | 0.876 | *P*=.245 | -2.737 to 0.699 |
| School vs Vocational school | -1.599 | 0.906 | *P*=.078 | -3.376 to 0.178 |
| **Income (RUB)** |  |  |  |  |
| Decline to answer vs <20,000 | -0.852 | 1.162 | *P*=.463 | -3.132 to 1.427 |
| 20,000-35,000 vs <20,000 | -0.53 | 0.702 | *P*=.45 | -1.908 to 0.847 |
| 35,000-70,000 vs <20,000 | -0.712 | 0.676 | *P*=.292 | -2.037 to 0.613 |
| 70,000-100,000 vs <20,000 | -0.192 | 0.772 | *P*=.803 | -1.705 to 1.321 |
| 100,000-150,000 vs <20,000 | -0.302 | 0.832 | *P*=.717 | -1.935 to 1.33 |
| 150,000+ vs <20,000 | -1.511 | 0.859 | *P*=.079 | -3.194 to 0.173 |
| **Chronic medical conditions** |  |  |  |  |
| Decline to answer vs No | 1.9 | 1.328 | *P*=.153 | -0.704 to 4.504 |
| Depression and (Cardiological or Respiratory) vs No | 1.525 | 1.448 | *P*=.292 | -1.314 to 4.365 |
| Depression or Neurological vs No | -0.056 | 1.067 | *P*=.958 | -2.148 to 2.035 |
| FoodAllergy/Rhinitis/Eczema/Psorias vs No | 0.969 | 0.679 | *P*=.154 | -0.362 to 2.301 |
| Cardiological vs No | 0.346 | 0.739 | *P*=.64 | -1.103 to 1.795 |
| Cardiological and Respiratory vs No | 1.23 | 2.497 | *P*=.622 | -3.666 to 6.127 |
| Renal/Hepatic/Diabetes vs No | 2.39 | 1.444 | *P*=.098 | -0.442 to 5.222 |
| Oncology/HIV vs No | -0.972 | 1.369 | *P*=.478 | -3.656 to 1.713 |
| Other vs No | 1.418 | 0.455 | *P*=.002 | 0.526 to 2.31 |
| Respiratory vs No | -0.246 | 1.407 | *P*=.861 | -3.005 to 2.514 |
| **Medications** |  |  |  |  |
| Neuroleptics/Antidepressant vs No | 1.392 | 0.911 | *P*=.127 | -0.395 to 3.178 |
| **Time spent on reading Covid news** |  |  |  |  |
| Decline to answer vs <30 mins | 10.748 | 3.466 | *P*=.002 | 3.951 to 17.544 |
| Do not follow vs <30 mins | -4.779 | 1.718 | *P*=.005 | -8.148 to -1.41 |
| Do not follow but they find me vs <30 mins | -0.574 | 0.706 | *P*=.417 | -1.958 to 0.811 |
| 30min-1h vs <30 mins | 2.412 | 0.464 | ***P*<.001** | 1.502 to 3.321 |
| 1-2h vs <30 mins | 3.885 | 0.584 | ***P*<.001** | 2.741 to 5.03 |
| 2-3h vs <30 mins | 4.744 | 0.858 | ***P*<.001** | 3.062 to 6.425 |
| 3h+ vs <30 mins | 6.796 | 1.043 | ***P*<.001** | 4.751 to 8.842 |
| **Smoking** |  |  |  |  |
| Former smoker vs Non-smoker | 0.648 | 0.501 | *P*=.196 | -0.334 to 1.63 |
| Current smoker vs Non-smoker | 1.148 | 0.429 | *P*=.008 | 0.307 to 1.989 |
| **Job Status** |  |  |  |  |
| Decline to answer vs Commute to work | 0.323 | 1.641 | *P*=.844 | -2.895 to 3.541 |
| Do not work vs Commute to work | -0.2 | 0.719 | *P*=.781 | -1.61 to 1.211 |
| Work from home vs Commute to work | -0.941 | 0.516 | *P*=.069 | -1.953 to 0.072 |
| Lost due to Covid and out of job vs Commute to work | 6.078 | 0.76 | ***P*<0.001** | 4.588 to 7.569 |
| **Healthcare-related job** |  |  |  |  |
| Medical student vs No | 1.64 | 2.374 | *P*=.49 | -3.016 to 6.296 |
| Volunteer/Hospital Management vs No | 0.839 | 1.869 | *P*=.653 | -2.825 to 4.504 |
| Nurse vs No | -1.818 | 2.92 | *P*=.534 | -7.544 to 3.908 |
| Physician vs No | -3.191 | 1.178 | *P*=.007 | -5.501 to -0.88 |
| **T-Anxiety** | 0.623 | 0.021 | ***P*<0.001** | 0.582 to 0.663 |
