# Supplementary material for: Excessive Media Consumption About COVID-19 is Associated With Increased State Anxiety: Outcomes of a Large Online Survey in Russia
Source: J Med Internet Res. 2020 Sep 11;22(9):e20955. doi: 10.2196/20955 (PMC7490003; doi:10.2196/20955)
Supplement: Multimedia Appendix 7 [file jmir_v22i9e20955_app7.docx]

**Table S5** Median scores reported by the respondents residing in different regions (with 40 respondents or more) of Russian Federation with regards to (a) Confidence in information and understanding. Combined median score on confidence in information and understanding is used (Q1. I feel informed about COVID-19; Q2. I feel informed about measures to prevent infection with COVID-19; Q3. I understand the guidance from healthcare authorities related to COVID-19) and (b) Combined median score on trust to state and local authorities and country readiness for pandemic (Q4. I think the country I am responding from is well prepared for COVID-19; Q5. I think all possible government measures to fight COVID-19 are being taken in my country; Q6. I think that all possible local authority measures to fight COVID-19 are being taken in my city/town/village/etc.; Q7. I trust the government in the country I am responding from; Q8. I trust the local authorities in the city/town/village/etc. I am responding from). Respondents were provided with a 9-point Likert scale, where 1 is ‘completely disagree’ and 9 is completely agree.

| **Area** | **Confidence in information and understanding** | **Trust to state and local authorities and country readiness for pandemic** |
| --- | --- | --- |
| **Altay** | 7.3 | 3.6 |
| **Arkhangel'sk** | 6.8 | 3.6 |
| **Astrakhan'** | 6.7 | 4.7 |
| **Bashkortostan** | 6.7 | 3.4 |
| **Belgorod** | 6.7 | 3.2 |
| **Bryansk** | 6.7 | 3.6 |
| **Chelyabinsk** | 7 | 3.8 |
| **Chuvash** | 7 | 3.9 |
| **City of St. Petersburg** | 7.3 | 3.1 |
| **Irkutsk** | 7.3 | 4.2 |
| **Ivanovo** | 7 | 3 |
| **Kaliningrad** | 7 | 3.4 |
| **Kaluga** | 7 | 4.4 |
| **Karelia** | 7 | 4.3 |
| **Kemerovo** | 7.3 | 3.8 |
| **Khabarovsk** | 7.3 | 3.6 |
| **Khanty-Mansiysk** | 7.3 | 4.4 |
| **Kirov** | 7.3 | 3.5 |
| **Komi** | 7.2 | 2.9 |
| **Kostroma** | 6.7 | 3.9 |
| **Krasnodar** | 7.3 | 4.2 |
| **Krasnoyarsk** | 7 | 3.6 |
| **Kursk** | 6.5 | 4 |
| **Leningrad Oblast’** | 7.3 | 4.1 |
| **Lipetsk** | 6.3 | 4.2 |
| **Mariy-El** | 7 | 2.8 |
| **Mordovia** | 7 | 3.8 |
| **Moscow City** | 7.3 | 4 |
| **Moskva** | 7 | 4 |
| **Murmansk** | 7.7 | 4.2 |
| **Nenets** | 7.3 | 4.8 |
| **Nizhniy Novgorod** | 7.3 | 4 |
| **Novosibirsk** | 7.3 | 3.8 |
| **Omsk** | 7.3 | 4 |
| **Orel** | 7.3 | 3.8 |
| **Orenburg** | 7.3 | 4 |
| **Penza** | 7 | 3.2 |
| **Perm'** | 7.3 | 4 |
| **Primor'ye** | 7.7 | 3.2 |
| **Rostov** | 7.3 | 3.8 |
| **Ryazan'** | 7 | 3.6 |
| **Sakha** | 7.7 | 4.4 |
| **Sakhalin** | 7.2 | 3.3 |
| **Samara** | 7.3 | 3.6 |
| **Saratov** | 7.3 | 3.2 |
| **Smolensk** | 7.5 | 4.2 |
| **Stavropol'** | 7.7 | 3.8 |
| **Sverdlovsk** | 7.3 | 3.8 |
| **Tambov** | 6.7 | 3.1 |
| **Tatarstan** | 7 | 4 |
| **Tomsk** | 7.7 | 3.6 |
| **Tula** | 7 | 3.4 |
| **Tver'** | 6.7 | 3.2 |
| **Tyumen'** | 7 | 5 |
| **Udmurt** | 7.3 | 4 |
| **Ul'yanovsk** | 7.3 | 2.9 |
| **Vladimir** | 7 | 3.4 |
| **Volgograd** | 7 | 3.6 |
| **Vologda** | 7 | 4.2 |
| **Voronezh** | 7 | 4 |
| **Yamal-Nenetsk** | 7.5 | 5 |
| **Yaroslavl'** | 6.7 | 3.2 |
