# Supplementary material for: Excessive Media Consumption About COVID-19 is Associated With Increased State Anxiety: Outcomes of a Large Online Survey in Russia
Source: J Med Internet Res. 2020 Sep 11;22(9):e20955. doi: 10.2196/20955 (PMC7490003; doi:10.2196/20955)
Supplement: Multimedia Appendix 8 [file jmir_v22i9e20955_app8.docx]

**Table S6** Regression model assessing associations between characteristics and state anxiety, including trust to state and local authorities and country readiness for pandemic and confidence in information and understanding

Statistically significant results presented in bold.

| **Model and variable** | **Coef.** | **Std. Err** | **P value** | **95% CI** |
| --- | --- | --- | --- | --- |
| **Sex** |  |  |  |  |
| Male vs Female | -4.298 | 0.22 | ***P*<.001** | -4.729 to -3.867 |
| **Age** | -0.005 | 0.01 | *P*=.632 | -0.025 to 0.015 |
| **Marital Status** |  |  |  |  |
| In relationship vs Single | -0.039 | 0.242 | *P*=.873 | -0.513 to 0.436 |
| Married vs Single | 0.117 | 0.225 | *P*=.602 | -0.323 to 0.557 |
| **Have children below the age of 18** |  |  |  |  |
| No vs Yes | -1.604 | 0.199 | ***P*<.001** | -1.993 to -1.215 |
| **Expecting a child** |  |  |  |  |
| No vs Yes | -1.003 | 0.333 | *P*=.003 | -1.655 to -0.351 |
| **Living in a capital** |  |  |  |  |
| No vs Yes | -0.659 | 0.152 | ***P*<.001** | -0.958 to -0.361 |
| **Education** |  |  |  |  |
| BSc vs Vocational school | 0.239 | 0.177 | *P*=.177 | -0.108 to 0.586 |
| MSc vs Vocational school | -2.009 | 0.895 | *P*=.025 | -3.763 to -0.255 |
| Other vs Vocational school | 0.327 | 0.276 | *P*=.236 | -0.213 to 0.868 |
| More than one degree vs Vocational school | -0.536 | 0.289 | *P*=.064 | -1.102 to 0.031 |
| Higher education in progress vs Vocational school | -0.147 | 0.286 | *P*=.608 | -0.707 to 0.413 |
| PhD vs Vocational school | -0.414 | 0.44 | *P*=.346 | -1.276 to 0.447 |
| School vs Vocational school | -1.963 | 0.5 | ***P*<.001** | -2.942 to -0.983 |
| **Income (RUB)** |  |  |  |  |
| Decline to answer vs. <20,000 | -0.326 | 0.359 | *P*=.364 | -1.03 to 0.378 |
| 20,000-35,000 vs <20,000 | -0.201 | 0.203 | *P*=.322 | -0.598 to 0.197 |
| 35,000-70,000 vs <20,000 | -0.438 | 0.206 | *P*=.033 | -0.842 to -0.035 |
| 70,000-100,000 vs <20,000 | -0.248 | 0.267 | *P*=.353 | -0.771 to 0.276 |
| 100,000-150,000 vs <20,000 | -0.267 | 0.326 | *P*=.414 | -0.906 to 0.373 |
| 150,000+ vs <20,000 | -0.619 | 0.372 | *P*=.096 | -1.349 to 0.11 |
| **Chronic medical conditions** |  |  |  |  |
| Decline to answer vs No | 2.47 | 0.566 | ***P*<.001** | 1.36 to 3.58 |
| Depression and (Cardiological or Respiratory) vs No | 2.794 | 0.648 | ***P*<.001** | 1.524 to 4.065 |
| Depression or Neurological vs No | 0.275 | 0.455 | *P*=.547 | -0.618 to 1.167 |
| FoodAllergy/Rhinitis/Eczema/Psorias vs No | 0.517 | 0.228 | *P*=.023 | 0.07 to 0.965 |
| Cardiological vs No | 0.687 | 0.377 | *P*=.068 | -0.052 to 1.426 |
| Cardiological and Respiratory vs No | 1.721 | 1.221 | *P*=.159 | -0.672 to 4.114 |
| Renal/Hepatic/Diabetes vs No | 1.043 | 0.383 | *P*=.006 | 0.293 to 1.793 |
| Oncology/HIV vs No | 0.789 | 0.61 | *P*=.196 | -0.406 to 1.984 |
| Other vs No | 1.114 | 0.161 | ***P*<.001** | 0.798 to 1.43 |
| Respiratory vs No | 0.325 | 0.742 | *P*=.661 | -1.13 to 1.78 |
| **Medications** |  |  |  |  |
| Neuroleptics/Antidepressant vs No | 1.187 | 0.367 | ***P=*.001** | 0.468 to 1.907 |
| **Time spent on reading Covid news** |  |  |  |  |
| Decline to answer vs <30 mins | 2.592 | 1.577 | *P*=.1 | -0.498 to 5.682 |
| Do not follow vs <30 mins | -4.813 | 0.549 | ***P*<.001** | -5.889 to -3.736 |
| Do not follow but they find me vs <30 mins | 0.912 | 0.221 | ***P*<.001** | 0.478 to 1.346 |
| 30min-1h vs <30 mins | 2.916 | 0.169 | ***P*<.001** | 2.584 to 3.247 |
| 1-2h vs <30 mins | 5.233 | 0.217 | ***P*<.001** | 4.807 to 5.658 |
| 2-3h vs <30 mins | 6.641 | 0.341 | ***P*<.001** | 5.973 to 7.309 |
| 3h+ vs <30 mins | 8.269 | 0.411 | ***P*<.001** | 7.464 to 9.075 |
| **Smoking** |  |  |  |  |
| Former smoker vs Non-smoker | 0.108 | 0.177 | *P*=.542 | -0.239 to 0.455 |
| Current smoker vs Non-smoker | 0.911 | 0.189 | **<0.001** | 0.541 to 1.281 |
| **Job Status** |  |  |  |  |
| Decline to answer vs Commute to work | 0.084 | 0.516 | 0.871 | -0.927 to 1.094 |
| Do not work vs Commute to work | -0.265 | 0.236 | 0.261 | -0.727 to 0.197 |
| Work from home vs Commute to work | -0.637 | 0.234 | 0.007 | -1.097 to -0.178 |
| Lost due to Covid and out of job vs Commute to work | 3.533 | 0.316 | **<0.001** | 2.914 to 4.152 |
| **Healthcare-related job** |  |  |  |  |
| Medical student vs No | -0.814 | 0.684 | 0.234 | -2.155 to 0.527 |
| Volunteer/Hospital Management vs No | -0.617 | 0.589 | 0.295 | -1.773 to 0.538 |
| Nurse vs No | -0.733 | 0.592 | 0.216 | -1.894 to 0.428 |
| Physician vs No | -0.768 | 0.341 | 0.024 | -1.436 to -0.1 |
| **T-Anxiety** | 0.514 | 0.008 | **<0.001** | 0.498 to 0.529 |
| **Confidence in information and understanding** | -0.65 | 0.038 | **<0.001** | -0.724 to -0.576 |
| **Trust to state and local authorities and country readiness for pandemic** | -0.731 | 0.034 | **<0.001** | -0.798 to -0.665 |
